# An Optimization Method of Ambiguity Function Based on Multi-Antenna Constrained and Application in Vehicle Attitude Determination

**DOI:** 10.3390/mi13010064

**Published:** 2021-12-30

**Authors:** Yinzhi Zhao, Jingui Zou, Peng Zhang, Jiming Guo, Xinzhe Wang, Gege Huang

**Affiliations:** 1 School of Geodesy and Geomatics, Wuhan University, Wuhan 430072, China; yzzhao_gnss@whu.edu.cn (Y.Z.); pzhang@sgg.whu.edu.cn (P.Z.); jmguo@sgg.whu.edu.cn (J.G.); wxz_gnss@whu.edu.cn (X.W.); 2 Beijing Key Laboratory of Urban Spatial Information Engineering, Beijing 100038, China; 3 Guangxi Key Laboratory of Spatial Information and Geomatics, Guilin University of Technology, Guilin 541004, China; 4 State Key Laboratory of Information Engineering in Surveying, Mapping and Remote Sensing, Wuhan University, Wuhan 430072, China; geehuang@whu.edu.cn

**Keywords:** GNSS, multi-antenna, attitude determination, ambiguity resolution, baseline-constrained AFM

## Abstract

The global navigation satellite system (GNSS)-based multi-antenna attitude determination method has the advantages of a simple algorithm and no error accumulation with time in long endurance operation. However, it is sometimes difficult to simultaneous obtain the fixed solutions of all antennas in vehicle attitude determination. If float or incorrect fixed solutions are used, precision and reliability of attitude cannot be guaranteed. Given this fact, a baseline-constrained ambiguity function method (BCAFM) based on a self-built four GNSS antennas hardware platform is proposed. The coordinates obtained by BCAFM can replace the unreliable real-time kinematic (RTK) float or incorrect fixed solutions, so as to assist the direct method for attitude determination. In the proposed BCAFM, the baseline constraint is applied to improve search efficiency (searching time), and the ambiguity function value (AFV) formula is optimized to enhance the discrimination of true peak. The correctness of the proposed method is verified by vehicle attitude determination results and baseline length difference. Experimental results demonstrate that the function values of error peaks are reduced, and the only true peak can be identified accurately. The valid epoch proportion increases by 14.95% after true peak coordinates are used to replace the GNSS-RTK float or incorrect fixed solutions. The precision of the three attitude angles is 0.54°, 1.46°, and 1.15°, respectively. Meanwhile, the RMS of baseline length difference is 3.8 mm.

## 1. Introduction

Accurate attitude measurement is an important technology in navigation, guidance, and control [1]. In general, the high-precision inertial navigation system (INS) with high cost can obtain the carrier attitude through mechanization. When Global Positioning System (GPS) went into service, some researchers proposed to use GPS multi-antenna (at least three receivers) to realize attitude determination. It is generally believed that carrier phase integer ambiguity resolution (AR) and the choice of the suitable method are the keys to high-precision attitude determination [2,3]. As long as the ambiguity is fixed correctly, the attitude can be determined based on the coordinates of the multi-antenna [4,5]. These two technologies have their strengths and weaknesses. INS has the advantages of strong anti-jamming ability and comprehensive information output. However, since the navigation results (velocity, position, and attitude) are obtained through integration, the system error will not be reduced but will accumulate with time and eventually affect its performance [6]. The positioning and attitude determination precision of GNSS multi-antenna is not affected by the duration of use. However, due to radio wave transmission and other factors, the precision is not high enough, and the operating range is limited. According to the above reasons, low-cost GNSS multi-antenna has outstanding advantages over INS if long endurance work is needed in low precision requirements situations such as vehicle attitude determination. It also has broad research and industrialization potential. More attention has been paid to the GNSS-based attitude determination method [7,8,9].

Attitude determination methods include the direct method, least square method, and optimal estimation method based on the Wahba problem [10,11]. There is not much difference between these methods in essence, and the precision is nearly the same. Many researchers have also studied the details. Among them, the ambiguity function method (AFM) and least-squares ambiguity decorrelation adjustment (LAMBDA) are considered as typical methods of search technique in coordinate and ambiguity domain respectively, which are widely used for GNSS-based attitude determination [12,13]. 

AFM was proposed by Counselman and Gourevitch (1981) [14] and then developed by Remondi (1990) [15], Han (1996) [16], Juang (1997) [17] and Caporali (2003) [18]. In recent years, it is also common to apply it for GNSS attitude determination. Yang et al. (2016) introduced the rotation matrix method to improve AFM and solve GNSS ambiguity resolution [19]. However, the efficiency of the ambiguity function and the problem of multi-peak are not discussed enough. Wang et al. (2019) improved AFM based on pitch-constrained and search candidates are greatly decreased [20]. The shortcoming of this method is lacking research on the search step. To address it, Cellmer (2021) proposed a new method of estimating the length of the search step [21]. The theory of the ambiguity function method has been further improved.

In the research of GNSS attitude determination based on LAMBDA, Teunissen et al. proposed CLAMBDA (Constrained LAMBDA) [22,23], they take the baseline length as constraint information to obtain the fixed solutions and attitude effectively. To enrich the related research of baseline constraint, Lu et al. (2019) conducted a detailed experimental analysis on the actual effect of CLAMBDA [24]. Liu et al. (2016) adopted the restriction of short baseline constraint in dual-frequency carrier phase ambiguity resolution [25]. Liu et al. (2018) presented an integrated attitude determination method based on an affine constraint [26]. Gong et al. also improved the LAMBDA method based on baseline vector constraint [27], but this method needs the assistance of other navigation equipment. Wu et al. (2020) reduced the GNSS-based attitude determination and positioning problems to a joint model with multivariable constraints [28]. However, the above attitude determination methods based on LAMBDA are unable to use when reliable fixed solutions cannot be obtained. Since the reliability of attitude solutions cannot be guaranteed, a novel method proposed by Li et al. (2018) improved attitude precision by 2.3° [29]. As in reference [30], these two manuscripts focus on improving attitude precision through quality control. When GNSS-RTK is unreliable, Zhang et al. (2020) proposed a method based on primary baseline switching to increase the availability of attitude determination [1]. However, this method cannot be used when more than two antennas in four antennas platforms are unavailable.

In addition to the above methods, some researchers have proposed other information-aided methods for attitude determination. For instance, some researchers consider using INS to supplement GNSS multi-antenna attitude determination. Cong et al. (2015) proposed a micro-electro-mechanical system (MEMS) INS-aided AR to optimize the GPS attitude determination results [31]. Zhu et al. (2019) fused GNSS multi-antenna and MEMS to acquire high accuracy attitude angles in GNSS challenged environments [13]. These solutions consider the supplement of MEMS INS when GNSS-RTK is unreliable, but the known relationship between GNSS antennas is not fully utilized.

To sum up, the current GNSS multi-antenna attitude determination methods do not fully explore the characteristics of multi-baseline; even if the least square method is applied, multi-baseline is only taken as redundant observation. Without the assistance of other sensors, GNSS attitude determination cannot be carried out when reliable fixed solutions of three or more antennas are not obtained, resulting in the loss of reliable results in vehicle attitude determination.

To solve this problem, a vehicle-mounted four-antenna platform is designed in this paper, in which the baseline length between antennas is known. Based on the platform, an ambiguity function method with additional baseline constraints is proposed to optimize the AFV, then reliable positions of four antennas can be obtained. As long as any one of the four antennas gets a reliable RTK fixed solution, the position of the other three antennas can be correctly calculated through BCAFM. Then the baseline vector is constructed for attitude determination. Valid epoch proportion and available attitude information can be increased through this method. As we all know, the two main difficulties that limit the application of AFM are efficiency and multi-peak [5]. Instead of the traditional AFM search method (sphere search based on a float or single point positioning (SPP) solution), a coordinate searching scope is conducted based on the position of another antenna with a reliable fixed solution. To accelerate the efficiency of AFM, the baseline length constraint is introduced and the loop search scope is established with “baseline length ± mean square error (MSE)” (antenna with fixed solution) as the radius. Besides, to verify the performance of BCAFM, the accuracy of coordinates corresponding to AFV peak is analyzed from both positioning results and attitude determination. The biggest contradiction in positioning is no truth value. In this manuscript, the difference between the known and calculated baseline length is used to verify, which can test the precision of positioning results. For attitude determination, the results of a high-precision SPAN-FSAS integrated navigation system based on tight coupling of GNSS and tactical IMU, are used as the reference for accuracy evaluations in this paper. Combined with four-antenna position distribution figures, baseline difference results, attitude results, and precision analysis, it can be proven that this method can effectively solve the problem of error peak in AFM, which has certain theoretical significance and practical value. BCAFM can also be transplanted to GNSS high-precision dynamic positioning. 

According to the investigation, the specific contributions are listed as follows:

(1) A four-antenna hardware platform is built, and a loop search method for matching the geometry of multiple antennas is proposed, which reduces the search range of AFM and greatly improves the calculation efficiency.

(2) An ambiguity function method with additional baseline constraints is proposed to optimize the calculation of AFV. The proposed method can enhance the discrimination between true peak and false peak by AFV optimization, and greatly improve the multi-peak problem of traditional AFM.

(3) The multi-antenna information has been fully applied and the baseline length truth value is introduced into the positioning and attitude determination for verification. The multi-antenna platform also helps to identify cases where ambiguity is incorrectly fixed.

(4) BCAFM can also effectively assist and supplement the positioning results of GNSS-RTK. The increase of attitude information can provide reliable positioning and attitude results in more epochs.

The remainder of the paper is organized as follows. Section 2 describes the detail of the proposed baseline-constrained ambiguity function method. Experimental results and analysis are illustrated in Section 3. Section 4 and Section 5 show conclusions and discussion.

## 2. Methodology

### 2.1. Attitude Determination Method Based on GNSS

#### 2.1.1. Double Difference Positioning Based on Carrier Phase

The function model of GNSS positioning can be expressed in the form of the following implicit functions [32]:

(1) $$O=F(X_{r},X_{s},\delta t_{r},\delta t_{s},\delta_{ion},\delta_{trop},\delta_{tide},\delta_{rel},N,\delta_{ref},\delta_{ref\_f},\varepsilon ,m)$$

where ***r*** and ***s*** indicate receivers and satellites, ***X_r_*** indicates the coordinates to be estimated, ***X_s_*** represents the coordinates of satellites. ***δt_r_*** and ***δt_s_*** are receiver and satellite clock errors. $\delta_{ion},\;\delta_{trop},\;\delta_{tide},\;\delta_{rel},\;\delta_{ref\_f}$ indicates the ionospheric error, tropospheric delay, tide, relativistic effects, and the frequency correction of relativistic effects. ***N*** is integer ambiguity, ***ε*** is noise, and ***m*** represents multipath effect. The carrier phase observations should be used in GNSS attitude determination to obtain high accuracy results. Since the baseline length of attitude determination is generally short, using the double-difference (DD) model [32], some errors such as clock, troposphere, ionosphere, and ephemeris errors can be eliminated or weakened. DD positioning formula is modeled as [33,34,35,36]:

(2) $$\nabla \Delta L_{br}^{ks}=\nabla \Delta \rho_{br}^{ks}+\nabla \Delta \varepsilon +\nabla \Delta m-\lambda \nabla \Delta N_{br}^{ks}$$

where ∇Δ is a DD operator. *k* and *s* indicate satellites. *b* and *r* represent base and rover stations. ***L*** indicates carrier phase observations. ***ρ*** is the geometric distance from satellites to receivers, and *λ* indicates the wavelength.

#### 2.1.2. Parameter Estimation and Ambiguity Resolution

To estimate parameters by Kalman filter, the state and observation equation of parameters are established:

(3) $$\begin{array}{l} X_{k}=\Phi_{k-1}X_{k-1}+\omega_{k-1}\\ Z_{k}=H_{k}X_{k}+v_{k} \end{array}$$

where *k* represents the current epoch; ***X*** represents the parameters to be estimated. ***Φ*** indicates transition matrix, and ***ω*** is process noise. ***Z*** is an observation matrix, and ***H*** is the coefficient matrix. ***v*** represents observation noise. Assuming that the system and observation noise is Gaussian white noise, the parameters have the following statistical characteristics:

(4) $$\begin{array}{l} E\left(\omega_{k}\right)=0,\;Cov\left(\omega_{k},\omega_{k}\right)=Q_{k}\\ E\left(v_{k}\right)=0,\;Cov\left(v_{k},v_{k}\right)=R_{k}\\ Cov\left(\omega_{k},v_{k}\right)=0 \end{array}$$

Given the initial information ***X*_0_** and ***P*_0_**, the formulas of the Kalman filter are shown as follow:

(5) $$\begin{array}{l} K_{k}=P_{k,k-1}H_{k}^{T}W_{k}\\ W_{k}={\left(H_{k}P_{k,k-1}H_{k}^{T}+R_{k}\right)}^{-1}\\ P_{k,k-1}=\Phi_{k-1}P_{k-1}\Phi_{k-1}^{T}+Q_{k-1},\;P_{k}=\left(I-K_{k}H_{k}\right)P_{k,k-1}\\ X_{k,k-1}=\Phi_{k-1}X_{k-1},\;X_{k}=X_{k,k-1}+K_{k}V_{k}\\ V_{k}=Z_{k}-H_{k}X_{k,k-1} \end{array}$$

where ***K****_k_* indicates Kalman gain, ***P****_k_* represents covariance matrix, and ***W****_k_* represents weight matrix. ***R****_k_* indicates the covariance matrix of observation noise, and ***Q****_k_* represents the covariance matrix of system process noise. ***X****_k,k−_*_1_ is the prediction matrix. ***V****_k_* is the innovation vector which means the residuals between observations and predictions. 

The coordinates change greatly between epochs in high dynamic positioning. Under the circumstances, the velocity parameter ***s*** is added to the state vector, and a uniform motion model is assumed. The state transition matrix and the dynamic process noise of the system are as follows:

(6) $$\Phi_{k-1}=\left(\begin{array}{ccc} I_{r} & I_{r}\tau_{r} & 0\\ 0 & I_{r} & 0\\ 0 & 0 & I_{q*q} \end{array}\right),\;Q_{k-1}=\left(\begin{array}{ccc} O_{r} & 0 & 0\\ 0 & Q_{s} & 0\\ 0 & 0 & O_{q*q} \end{array}\right)$$

where $Q_{s}=I_{r}^{T}diag(\sigma_{sx}^{2}\tau_{r},\;\sigma_{sy}^{2}\tau_{r},\;\sigma_{sz}^{2}\tau_{r})I_{r}$, $\left(\sigma_{sx}^{2},\;\sigma_{sy}^{2},\;\sigma_{sz}^{2}\right)$ is the process noise component of the velocity on the *x*, *y*, and *z* axes. *q* represents the number of DD ambiguities. To avoid the divergence caused by the inaccuracy motion model in Kalman filter processing, the weight can be dynamically adjusted to realize a robust Kalman filter [37].

The DD ambiguities float solutions and covariance matrix can be obtained by the above parameter estimation. The classic LAMBDA is used to search and fix the ambiguities; then, the high precision fixed solutions will be acquired.

#### 2.1.3. Attitude Determination

The baseline vector is constructed based on the positioning results when the coordinates of four antennas are obtained to carry out attitude determination. GNSS attitude determination is generally based on the coordinate transformation principle. We adopt the direct method in which the projection of baseline vector in body frame (b-frame) and local navigation frame (n-frame) are used. The coordinate conversion matrix between them is applied to calculate the attitude angles. Only the baseline vector projection under n-frame is necessary for the direct method. If the fixed solutions of three antennas (two baselines) are obtained, three angles can be calculated.

**Figure 1:**
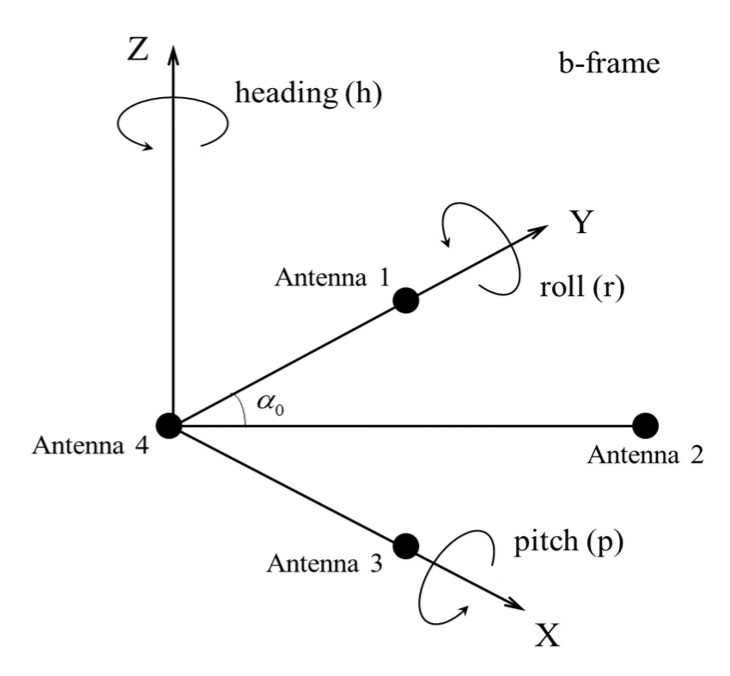
Schematic diagram of four-antenna.

According to the fixed solutions of each antenna, the antenna corresponding to the minimum posterior mean square error is taken as the primary antenna. For example, antenna 4 shown in Figure 1 is regarded as the primary antenna, and antenna 1, 2 can also obtain the fixed solutions. Baseline 1-4 is regarded as the primary baseline on this premise. The subsidiary baseline 4-2 and baseline 4-1 are not collinear with a known angle *α*_0_.

Since the baseline lengths $l_{4}^{1}$, $l_{4}^{2}$ and *α*_0_ are known information, the baseline vectors projections *X*_1_*^b^*, *X*_2_*^b^* under b-frame are easy to obtain. Through GNSS-RTK, the baseline vectors projection *X*_1_*^n^*, *X*_2_*^n^* under n-frame are also obtained. According to the relationship between *X^b^* and *X^n^*, three attitude angles can be derived as:

(7) $$\begin{array}{c} h=\arctan\left(\frac{e_{41}}{n_{41}}\right)\\ p=\arctan\left(\frac{u_{41}}{\sqrt{e_{41}^{2}+n_{41}^{2}}}\right)\\ r=-\arctan\left(\frac{-\sin p\sin he_{42}-\sin p\cos hn_{42}+\cos pu_{42}}{\cos he_{42}-\sin hn_{42}}\right) \end{array}$$

where *h*, *p*, and *r* are heading, pitch, and roll angles, respectively. However, the most troublesome problem is that the attitude determination results have a great deviation when the reliable fixed solutions cannot be obtained.

### 2.2. Improved Baseline-Constrained AFM for Attitude Determination

#### 2.2.1. Introduction of AFM

As mentioned in Section 1 and the previous section, it is impossible to guarantee that reliable fixed solutions of four antennas can be obtained all the time. In this manuscript, AFM is introduced to search the antenna position when the reliable fixed solutions cannot be obtained. AFM is a search method based on the coordinate domain and does not explicitly calculate the ambiguity but directly uses the integer characteristics of ambiguity [38]. The formula of AFM based on the DD carrier phase follows:

(8) $$AFV(X_{c},Y_{c},Z_{c})=\frac{1}{n}\sum_{i=1}^{n}\cos2\pi (\varphi_{real}^{}[E_{base}|E_{rover}]-\varphi_{cal}^{}[E_{base}|X_{c},Y_{c},Z_{c}]\;)$$

where (*X_c_*, *Y_c_*, *Z_c_*) indicates candidates in search space. *n* indicates a common satellite between base and rover stations. *φ_real_*[*E_base_|E_rover_*] are the DD observations. *φ_cal_*[*E_base_|X_c_*, *Y_c_*, *Z_c_*] represents DD observations calculated from the candidates.

The two disadvantages of AFM are efficiency and multi-peak. There are mainly two reasons for low efficiency: one is that the search scope depends on the positioning accuracy of the rover station. Once the ambiguities of the rover station cannot be fixed (only float or SPP solutions), the search scope will be large, and it is not easy to determine the specific range; the other is that the coordinate accuracy obtained by AFM depends on search steps. The smaller the step length, the higher the positioning precision of the rover station; however, the search efficiency will also be reduced. In addition, it is easy to understand the multi-peak problem since the characteristic of a cosine function. Theoretically, the AFV will be equal to 1 as long as the ambiguity is an integer. Considering the influence of noise and multipath, the largest peak value will be close to 1. Aiming at the above difficulties, the core idea of this article is to solve them through multi-baseline constraints.

#### 2.2.2. Search Scope Optimization of AFM Based on Known Baseline-Constrained

To reasonably determine and reduce the search range of AFM to a certain extent, search scope is established at a fixed solution. Since the length between any two antennas is known, the search radius can be set to this known length in theory. Considering noise and positioning error of the antenna with coordinate fixed solutions, search scope can be limited as follows:

(9) $$scope={\left\{p_{i}\left|l_{i}^{j}-\kappa \sigma_{j}\leq norm\left(p_{i}-p_{j}\right)\right.\leq l_{i}^{j}+\kappa \sigma_{j}\right\}}_{step=\sigma_{j}},\kappa \geq 3$$

where *scope* means search scope. *i* and *j* indicate float and fixed solution antenna, respectively. **p***_i_* indicates position vector candidates of float solution antenna. $l_{i}^{j}$ is known baseline length. **p***_j_* indicates the position vector of the fixed solution. *σ_j_* is the posterior mean square error of the fixed solution antenna. *step* represents the search step.

#### 2.2.3. AFV Optimization and Error Peak Elimination Based on Known Baseline-Constrained

Another baseline is introduced as a constraint with regard to the problem of error peaks which is difficult to solve in AFM. In the search scope described in the previous section, AFVs corresponding to each position candidate and the baseline length from those candidates to another antenna are calculated. If a candidate is close to a real position, the difference between calculated and truth length is small. Therefore, the difference between baseline length calculated from each candidate position and known value is used as a constraint to identify the true peak. The direct idea is to divide AFVs of each position candidate by these differences and then obtain the coordinate corresponding to the maximum function value. Although this method can remove some error peaks, it is also possible to enlarge many peaks to a large abnormal value, which is not conducive to the identification of true peak. In this manuscript, the exponential function based on the natural constant **e** is used as a denominator, and the independent variable is the absolute value of the baseline length difference. The advantage is that when the baseline length difference is small, the function value of the exponential function will be close to 1, which ensures that the truth peak will not be covered by some pseudo peaks with a small baseline length difference but small AFV. Once the difference of baseline length is slightly larger, the exponential function value will increase rapidly, and the AFV based on baseline-constrained will be greatly reduced, leading to a reduction of error peaks. Finally, the true peak with the largest AFV and a small baseline length difference will be retained. This method is particularly effective when the data quality of the float solutions antenna is poor since the true peak here is covered by a large number of peaks. To sum up, the AFV based on baseline-constrained is shown as follows.

(10) $$BCAFV=AFV/\exp\left\{abs\left[norm(p_{i}-p_{m})-l_{i}^{m}\right]\right\}$$

**Figure 2:**
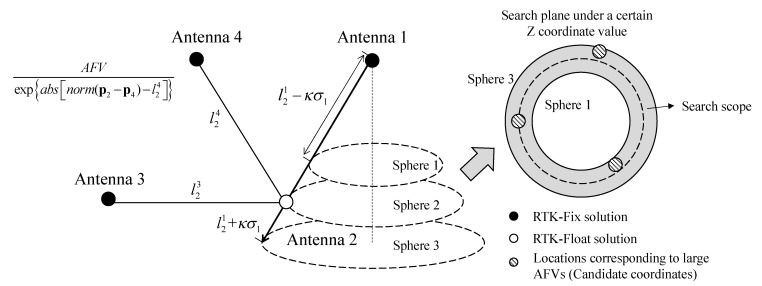
Schematic diagram of BCAFM.

To represent the above process, take antenna 1, 3, 4 with a fixed solution and antenna 2 with a float solution as an example (*i* = 2, *j* = 1, *m* = 4) and draw the following schematic Figure 2.

In Figure 2, the position of antenna 2 only obtained RTK float solutions. Taking antenna 1 as the center and baseline $l_{2}^{1}\pm \kappa \sigma_{1}$ as the radius, a search scope around antenna 2 is constructed. Meanwhile, the cross-section of the search scope is a loop. Then the AFVs are calculated based on position candidates. For each AFV obtained, the baseline length is calculated from its corresponding coordinates and the position of antenna 4. Finally, according to formula (10), AFV can be modified to BCAFV and its corresponding coordinates are taken as the position candidates of antenna 2.

If the last antenna (such as antenna 3 in Figure 2) can obtain RTK fixed solution or its accurate coordinates have been obtained through the above method, the last baseline length can be used for verification:

(11) $$\begin{array}{l} d_{i}^{n}=abs\left[norm\left(p_{i}\left|{}_{BCAFV-peak}\right.-p_{n}\right)-l_{i}^{n}\right]\\ p_{i}=\left\{\begin{array}{ll} p_{i}{|}_{BCAFV-peak} & d_{i}^{n}\leq T\\ p_{float} & d_{i}^{n}>T \end{array}\right. \end{array}$$

where *T* indicates the threshold of the baseline length difference. If combined with Figure 2, $d_{i}^{n}$ indicates the absolute value of length difference of baseline 3-2 and $l_{i}^{n}$ represents the known length of baseline 3-2.

### 2.3. Flow Chart of Improved Attitude Determination Algorithm

**Figure 3:**
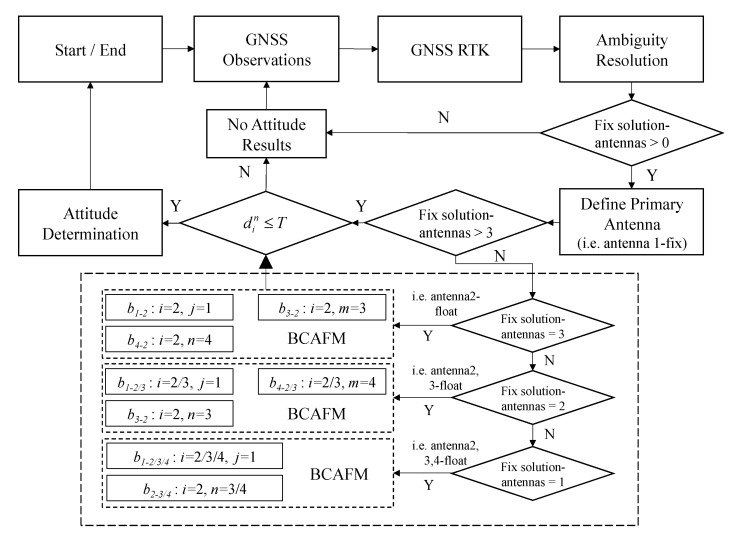
The workflow of the proposed method.

In summary, three independent baselines are applied to BCAFM as a constraint. The first baseline is mainly used to help establish the search scope of AFM and improve the search efficiency; the second baseline is mainly used to improve the calculation method of AFV to eliminate error peaks; the third baseline is used to verify the correctness. However, if there is only one antenna in four acquires coordinate fixed solution, only standard AFM is used to obtain another antenna position. The proposed method can be carried out. Combined with the attitude determination process, the proposed method can be represented by the following flow chart Figure 3:

## 3. Experiment

### 3.1. Introduction of the Experimental Platform

**Figure 4:**
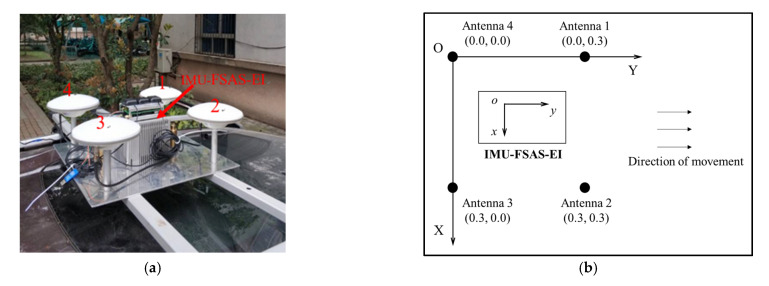
The four-antenna platform: (**a**) Real photos; (**b**) schematic diagrams.

To test BCAFM, a GNSS multi-antenna platform is designed. Four GPS500 antennas are fixed on a steel plate to form a 0.3 m×0.3 m rectangle which is shown in Figure 4. GPS500 can receive signals of GPS L1/L2, BDS (Beidou Navigation Satellite System) B1/B2/B3. As described in Section 1, the proposed method will be verified by the results of the attitude determination and baseline length. The results of attitude determination are compared with a high precision integrated navigation device, SPAN-FSAS produced in Novatel. The nominal precision of pitch, roll, and azimuth angles are 0.015°, 0.015°, and 0.041°.

The four-antenna platform was mounted on the roof of an automobile. We drove along Bayi Road which is located in Wuhan, China, for about 15 min. The sampling interval is 1 second which is relatively low for dynamic attitude determination but sufficient for GNSS positioning applications. The traffic condition in the first half was smooth, and the driving speed was fast, while the second half was slow.

### 3.2. Performance Analysis of BCAFM

During the whole experiment, the RTK Positioning results of four antennas consist of the following five situations:

Case 1: four antennas with the correct coordinate fixed solution;

Case 2: three antennas with the fixed solution and one antenna with a float solution;

Case 3: two antennas with the fixed solution; 

Case 4: one antenna with a fixed solution; 

Case 5: all four antennas with the float solution or incorrect fixed solution.

The number of epochs in five situations is shown in Table 1.

Whether the antenna can get the correct coordinate fixed solution is also determined by setting the threshold of baseline length residuals. If the baseline length calculated by the fixed solution is obviously not equal to the known value, it means that the fixed solution is wrong. Under these circumstances, attitude determination is also unreliable. Therefore, all attitude determination methods including the proposed method is not applicable to Case 5 in Table 1. When the observations quality of all antennas is poor, AFM cannot search the correct coordinates either. In order to solve this problem, an inertial navigation system or other sensors need to be integrated, but this is not the focus of this manuscript. 

The traditional GNSS attitude determination method can only output reliable attitude results in Case 1 and 2. If the direct method is adopted, only Case 1 can be handled when an antenna in the primary baseline cannot obtain the correct RTK fixed solutions. But the proposed BCAFM can deal with Cases 1–4. The correctness and feasibility of BCAFM will be verified in detail in the next sections.

#### 3.2.1. Search Efficiency Analysis

**Figure 5:**
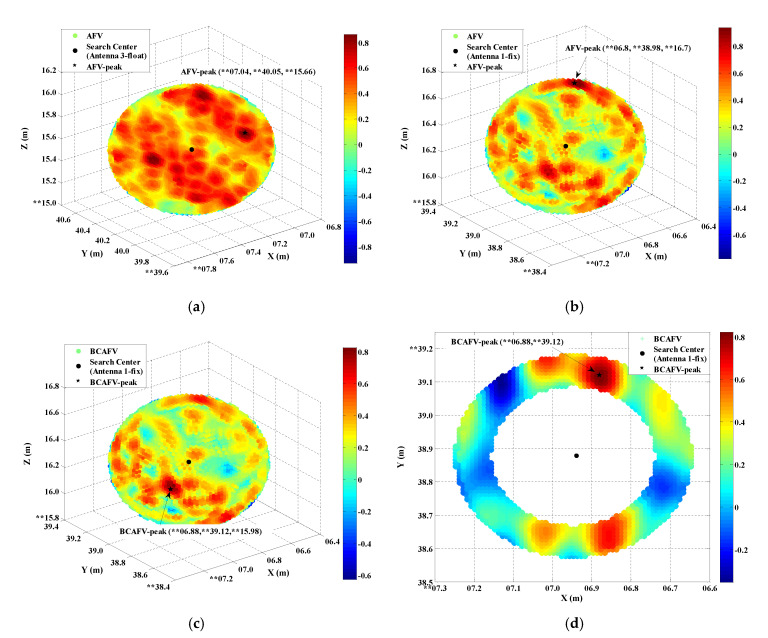
The search candidates of AFM at epoch 03:03:31. (**a**) Search scope of traditional AFM based on the float solution of antenna 3. (**b**) Search scope of AFM based on the fixed solution of antenna 1. (**c**) Search scope of BCAFM. (**d**) The cross section of search scope in (**c**) under the Z coordinate component of peak value. “**” in the figures represents the first few digits in the coordinates what are hidden for the confidentiality of the coordinates.

The following Figure 5 shows the search candidates of AFM at epoch 03:03:31. In this epoch, antenna 1, 2, and 4 can obtain reliable fixed solutions, only antenna 3 obtained coordinate float solutions. Figure 5a is the search scope based on the float solution of antenna 3, and the search step is 0.02m. Since there is no prior value, the search radius and step length are given with certain randomness. In Figure 5b, the fixed solution of antenna 1 is taken as search center, and the mean square error of antenna 1 is taken as search step. The difference between Figure 5b,c is that the baseline constraints described in Section 2.2 are introduced in the calculation of the AFVs in Figure 5c. Figure 5d is the cross-section of the search scope in Figure 5c under the Z coordinate component of the peak value. The section forms a loop, which is consistent with the theory in Section 2.2. The coordinate system in Figure 5 is a geocentric coordinate system. For the confidentiality of coordinates, the first few digits are hidden.

Compared with Figure 5a,b, the search density of Figure 5a is higher, and the number of points with large AFVs is too many, so it is difficult to accurately identify the maximum AFV. Moreover, whether the AFV peak in Figure 5a is a true peak cannot be guaranteed due to the randomness of search radius and step length. In contrast, the search density of Figure 5b is greatly reduced, and the number of points with large AFVs is also greatly decreased. According to Figure 5b,c, it can be found that compared with AFVs, BCAFVs are much smaller than AFVs, which makes the BCAFV peak (the largest value) more significant. However, the coordinates corresponding to the peaks of AFV in Figure 5b and BCAFV in Figure 5c are not consistent. Whether the two peaks are true will be explained in the next section. In addition, compared with traditional AFM in Figure 5a, BCAFM transforms search scope from a sphere to a loop, and has a reliable radius and step length acquisition way. Therefore, BCAFM will be significantly better than traditional AFM in efficiency. The following Table 2 shows the search time of AFM and BCAFM in three sample epochs.

According to Table 2, it is obvious that the efficiency of BCAFM is greatly improved compared with traditional AFM, and the search time is less than 0.5s. This is of great significance for real-time attitude determination. Combined with Figure 5, Table 2 and correlation analysis, traditional AFM cannot be used in vehicle attitude determination. The results of traditional AFM will not be presented later in the following sections.

#### 3.2.2. Search Results Analysis

The key of BCAFM is to find the correct antenna position. In this section, we will select three epochs from Case 2, 3, and 4 in Table 1 for verification. The ambiguity resolutions of four antennas in the selected three epochs are shown in Table 3: 

According to the analysis of Figure 5a and Section 3.2.1, the way to establish AFM search scope based on the antenna with coordinate float solutions has low efficiency and disordered peaks distribution. Moreover, since the float solution is not near the truth value, the search scope and step length cannot be accurately assigned. This situation makes the corresponding AFV peak unreliable. Therefore, it is more reasonable to use other antennas with fixed solutions as the center and the known baseline length (± MSE) as radius for searching. The AFVs in the following tables are all obtained in this way.

Table 4 lists some large function values (as shown in Figure 5b,c) and corresponding coordinates in the 03:03:31 epoch, which belongs to Case 2. AFM (based on antenna 1 with fixed solutions) and BCAFM are applied, respectively. At this time, the search center is antenna 1 with fixed solutions, and the search radius is the length of baseline “1–3” ± MSE of antenna 1. Similarly, for the confidentiality of coordinates, the first few digits are hidden.

**Figure 6:**
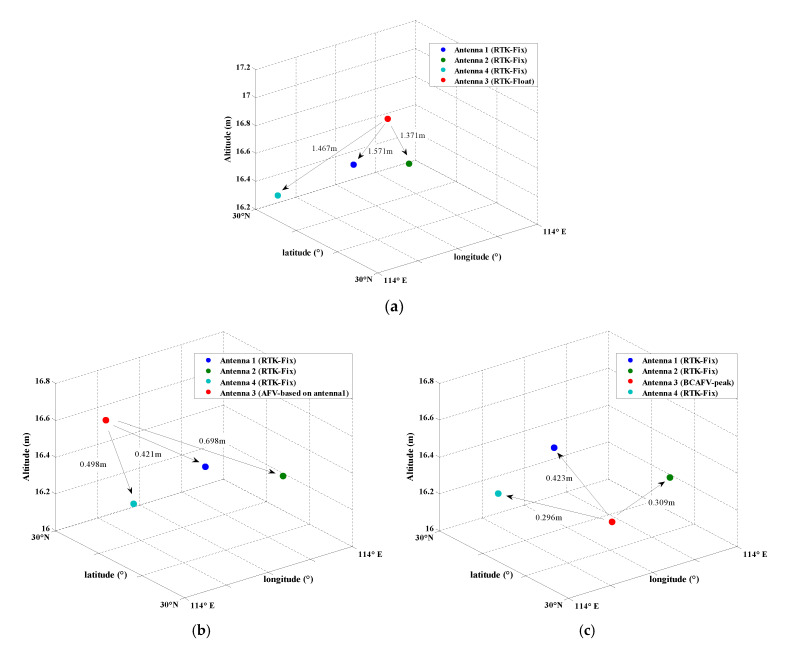
Spatial distribution of four antennas in different positioning modes. (**a**) Antennas 1, 2 and 4 are RTK fixed solution positions, and antenna 3 is float solution position. (**b**) Antenna 3 is Peak1 obtained by AFM in Table 4, also the peak in Figure 5b. (**c**) Antenna 3 is Peak2 obtained by BCAFM in Table 4, also the peak in Figure 5c.

According to Table 4, the maximum AFV is recorded as Peak1 and the maximum BCAFV is recorded as Peak2. Combined with Table 4 and Figure 5b,c, the peak obtained by the AFM method is not significant, and some other large AFVs interfere with the judgment. The peak of BCAFM is relatively independent. Even for other large BCAFVs, the corresponding coordinates are also near the peak. Figure 6 shows the spatial distribution of four antennas in different positioning modes. In Figure 6a, antennas 1, 2, and 4 obtained RTK fixed solutions. Antenna 3 can only obtain float solutions; In Figure 6b, the position of antenna 3 is Peak1 obtained by AFM in Table 4, which is also the peak in Figure 5b; In Figure 6c, the position of antenna 3 is Peak2 obtained by BCAFM in Table 4, which is also the peak in Figure 5c.

According to Figure 6, it can be found that only the positions of four antennas in Figure 6c conform to the known configuration. It can be considered that the correct truth peak is found by BCAFM while other methods cannot get the accurate position of antenna 3. Figure 6b also shows that error peaks and true peaks cannot be distinguished without the baseline constraint when the data quality is poor, and then correct coordinates of the antenna cannot be obtained. The accuracy of coordinates corresponding to the BCAFV peak will be further verified by baseline length residuals and attitude determination results.

**Figure 7:**
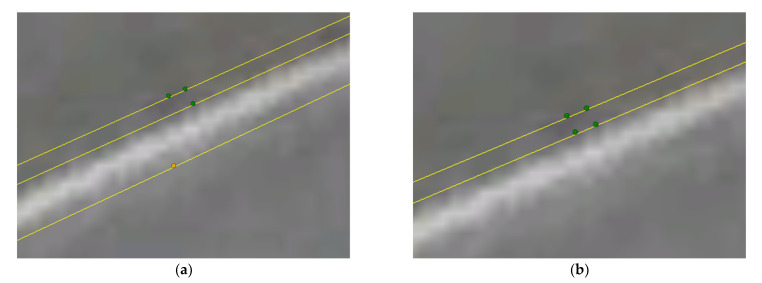
Distribution of four antennas on Google Earth (GPST 03:03:31): (**a**) Result of RTK; (**b**) Result of BCAFM.

Figure 7 shows the location distribution of four antennas on Google Earth (GPST 03:03:31) when RTK and BCAFM are applied respectively. It can also be found from the images that the antenna location obtained by BCAFM is more reliable.

In Case 3, we choose the epoch (GPST 03:05:15) in which antennas 2 and 3 cannot obtain the coordinate fixed solutions. BCAFM is used to search the positions of two antennas based on baseline 1-3 and 1-2, respectively. Taking baseline 1-3 as an example, the following table lists the relatively large function values and corresponding coordinates when AFM (AFM based on antenna 1) and BCAFM are used at 03:05:15.

According to Table 5, the AFV peak is still relatively large after baseline constraint in this group of data, indicating that the true peak (Peak1) is not covered by error peaks. However, there is still a dilemma that the function value is large (Peak2) but the corresponding coordinates are not near the true peak when using AFM, which also brings some difficulties for the recognition of true peak. Therefore, there is no sufficient reason to regard the maximum AFV as a true peak. After adopting BCAFM, although the peak coincides with AFM, this method can greatly reduce function values of other error peaks (such as Peak2), causing the true peak more significant.

**Figure 8:**
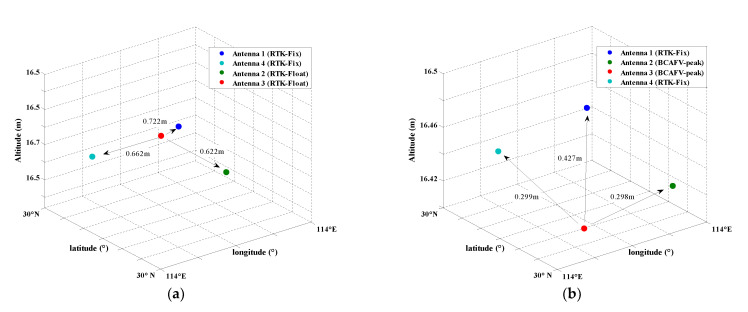
Spatial distribution of four antennas in different positioning modes. (**a**) Antennas 1 and 4 are RTK fixed solution positions and antennas 2 and 3 are float solution positions. (**b**) Antennas 2 and 3 are the positions corresponding to the truth peak.

Since the largest peak of AFM and BCAFM coincide, Figure 8 only shows two circumstances, which are different from Figure 6. In Figure 8a, antennas 1 and 4 obtained RTK fixed solutions, and antennas 2 and 3 only obtained float solutions; In Figure 8b, antennas 2 and 3 are the positions corresponding to the true peak obtained by BCAFM in Table 5.

**Figure 9:**
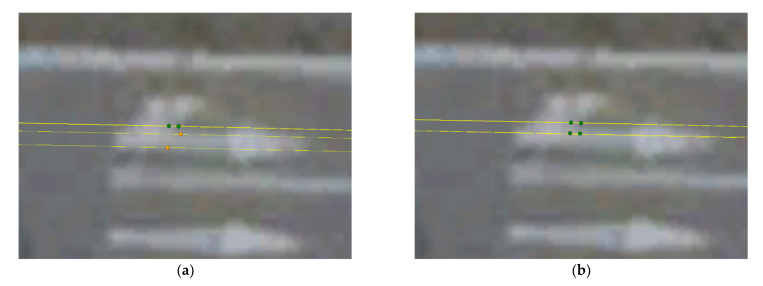
Distribution of four antennas on Google Earth (GPST 03:05:15): (**a**) Result of RTK; (**b**) Result of BCAFM.

Figure 9 shows the location distribution of four antennas on Google Earth (GPST 03:05:15) when RTK and BCAFM are used, respectively. We can also learn from the images that the antenna location obtained by BCAFM is more reliable.

In case 4, we choose the epoch (GPST 03:03:37) in which antennas 2, 3, and 4 cannot obtain the coordinate fixed solutions. BCAFM is used to search the positions of two antennas based on baseline 1-3 and 1-2 respectively, meanwhile, the known length of baseline 2-4 is taken as a constraint. Then the position of antenna 3 is searched based on baseline 1-3, and the known baselines 3-2 and 3-4 are taken as constraints and verification conditions.

Similarly, taking baseline 1-3 as an example, the following Table 6 lists the relatively large function values and corresponding coordinates when AFM (AFM based on antenna 1) and BCAFM are used at 03:03:37 epoch.

According to Table 6, the AFV peak is still relatively large after baseline constraint in this group of data, indicating that the truth peak (Peak1) is not covered by error peaks. Furthermore, there is no other peak with a large function value to affect the identification of the true peak. Therefore, it can be considered that Peak1 is the true peak.

**Figure 10:**
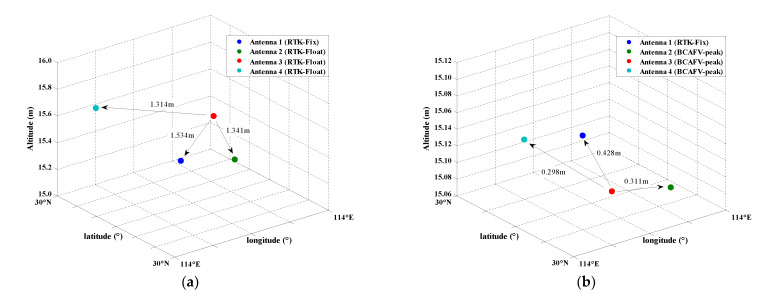
Spatial distribution of four antennas in different positioning modes. (**a**) Antenna 1 is the fixed solution position of RTK, and antennas 2, 3 and 4 are all float solution positions. (**b**) Antenna 2, 3, and 4 are all the positions searched by BCAFM and the position of antenna 3 is the coordinate corresponding to the peak value.

Different from Figure 6 and Figure 10 also only show two circumstances since the largest peak of AFM and BCAFM coincide. In Figure 10a, antenna 1 obtained the fixed solutions of RTK, and antennas 2, 3, and 4 all obtain float solutions; In Figure 10b, the positions of antenna 2, 3, and 4 are all searched by BCAFM, and the position of antenna 3 is the coordinate corresponding to the peak value obtained by BCAFM in Table 6. The peak of AFM and BCAFM are the same at this epoch, and this peak is significant.

**Figure 11:**
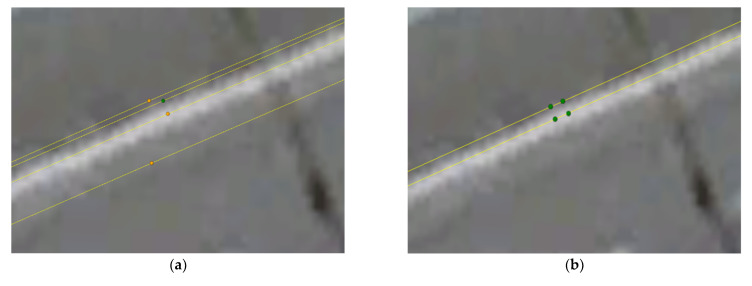
Distribution of four antennas on Google Earth (GPST 03:03:37): (**a**) Result of RTK; (**b**) Result of BCAFM.

Figure 11 shows the location distribution of four antennas on Google Earth (GPST 03:03:37) when RTK and BCAFM are used, respectively. We can also learn from the images that the antenna location obtained by BCAFM is more reliable.

In this section, the calculated baseline length and spatial position distribution of four antennas are used to preliminarily verify that the peak obtained by BCAFM is true. Besides, the coordinates corresponding to these peaks are close to the truth value, which indicates that positions of the antennas with float solutions can be correctly calculated through BCAFM. Since the biggest challenge of positioning is no truth value for verification, the precision of BCAFM will be further proved by combining the baseline length truth value and attitude determination results.

### 3.3. Analysis of Attitude Determination Results and Baseline Length Residuals

**Figure 12:**
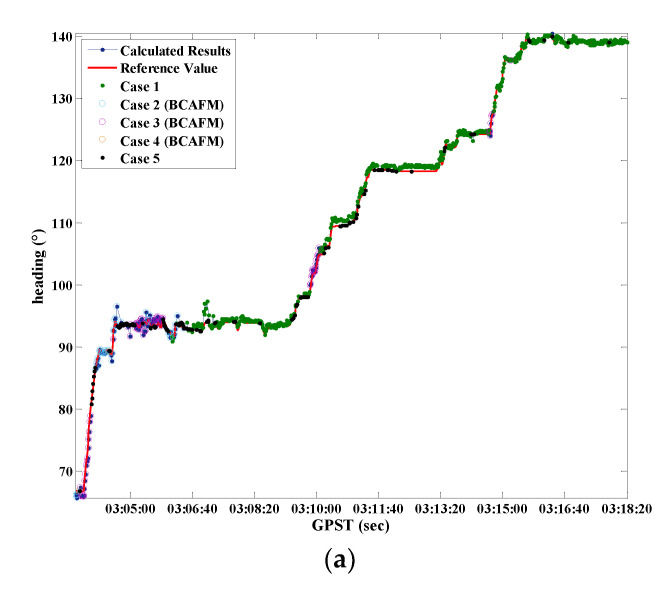

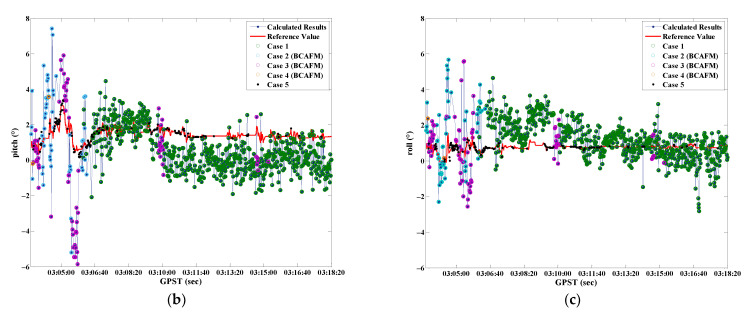
Three attitude angles calculated by BCAFM and the reference values. (**a**) Heading; (**b**) Pitch; (**c**) Roll.

The overall attitude determination is based on RTK fixed positioning results, and three angles are calculated by the direct method (Formula (7)). When only RTK float or incorrect fixed solutions (Case 2, 3, 4) can be obtained for some antennas, BCAFM is used to assist in obtaining the antenna coordinates, so as to supplement attitude determination results. Heading, pitch, and roll angles are shown in Figure 12a–c. The blue curve represents the results calculated by the direct method with the assistance of BCAFM. The red line indicates the reference value. The epochs of Case 1, 2, 3, 4, 5 are distinguished in different colors. The hollow circle in Figure 12 represents the epoch when adopting BCAFM.

According to Figure 12, the deviation between the calculated results and reference values is small. Especially for the most concerned heading angle in vehicle attitude determination, the calculated results are basically consistent with the reference value. We can learn from Figure 12 that most of the epochs adopting BCAFM are concentrated in the front of the route. The reason may be the multipath effect of surrounding buildings and lakes or tree shelters. As a result, the data quality is affected to a certain extent and some antennas cannot get the coordinate fixed solutions. Although the data quality also leads to small AFVs indeed, error peaks are effectively eliminated, and the true peak can be identified through BCAFM. This method improves the availability of data to a certain extent, so that attitude results can be provided in more epochs.

In this manuscript, we define the epochs when four antennas obtain coordinate float or incorrect fixed solutions as invalid epoch (also Case 2, 3, 4, 5 in Table 1). It is considered that the attitude results of these epochs are unreliable. There are 616 epochs in which all four antennas can obtain reliable fixed solutions during the experiment. With the assistance of BCAFM, the number of valid epochs increased to 749, and the proportion is increased from 69.21% to 84.16%. These results mean that more attitude information can be output in the experiment. In order to present the unreliability of the RTK float solution for attitude determination, the attitude precision based on RTK (fixed and float) and BCAFM are listed in Table 7 below.

**Figure 13:**
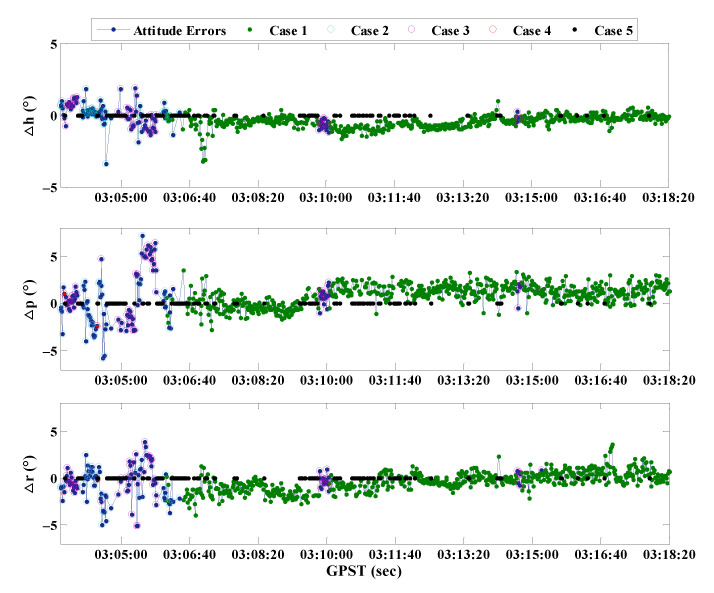
Attitude determination errors obtained by the assistance of BCAFM.

According to Table 7, it can be considered that the results of attitude determination based on RTK float solutions have a large deviation, which is consistent with the previous theory, indicating that attitude determination based on RTK coordinate float solutions is unreliable. Besides, the precision is high while adopting BCAFM instead of float solutions, especially the heading which is most concerned about in-vehicle attitude determination can reach 0.54°. Figure 13 shows the determination errors of three attitude angles obtained by the assistance of BCAFM, Δh, Δp, and Δr are the errors of three attitude angles, respectively. The epochs of Case 1, 2, 3, 4, 5 are distinguished in different colors.

According to Figure 13, a few epochs with large errors are concentrated in the period when BCAFM is needed to assist positioning. This phenomenon indicates that although BCAFM can obtain the correct position of the antenna, the precision will still be affected to a certain extent due to noise, multipath, and other factors. In addition, the precision of pitch and roll angles is worse than the heading angle. The reason is that the calculation of heading angle only depends on the baseline vector projection under the n-frame. However, the calculation of pitch and roll angles involves power and square root operation, which will also amplify the noise and reduce the calculation precision. 

**Figure 14:**
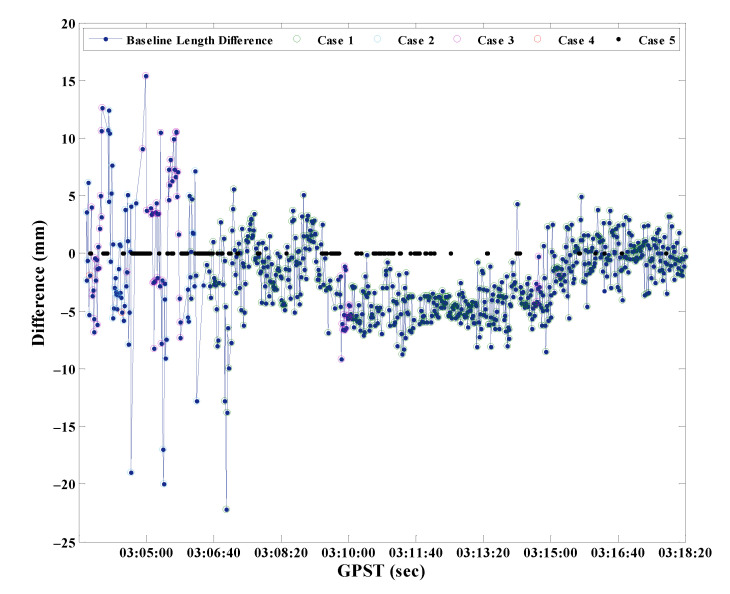
The length difference of baseline 1-3 between the known and calculated values.

At last, after the coordinates float solutions are replaced by BCAFM, the length difference of baseline 1-3 between the known and calculated values is shown in Figure 14. The epochs of Case 1, 2, 3, 4, 5 are distinguished in different colors. Since the known baseline length is a priori truth value, it can also illustrate the correctness of coordinates obtained by BCAFM. The RMS of baseline residuals in Figure 14 is 3.8 mm.

The known length of baseline 1-3 is 0.424 m. According to Figure 14, the baseline length difference between the known value and calculated value by BCAFM is maintained within 20 mm, which indicates that the accuracy of the coordinates calculated by BCAFM can be equivalent to that of RTK fixed solutions. Furthermore, it is proved that BCAFM can obtain the true peak while ensuring search efficiency. The coordinates corresponding to the true peak are near the truth value.

## 4. Conclusions

We realize a multi GNSS antenna vehicle positioning and attitude determination algorithm based on a self-built four antenna platform. Since reliable coordinate fixed solutions cannot always be acquired in vehicle attitude determination and positioning, an improved ambiguity function method based on multi-baseline constrained (BCAFM) is proposed. Firstly, the loop search scope (replacing the sphere with loop) is established by taking the antenna with a coordinate fixed solution as searching center and the first known baseline length as the radius. Secondly, the second known baseline is used as a constraint condition to improve and optimize the calculation of AFV. Finally, if there are more known baselines, they can be used as verification conditions. The proposed method can solve the low calculation efficiency and multi-peak problems of AFM. The experimental results show that the single epoch search time of BCAFM is less than 0.5 s. In addition, the optimized BCAFV peak is more prominent than error peaks since the function values of error peaks are greatly reduced, thus the accuracy and reliability of true peak identification are improved. Compared with traditional AFM and RTK float solutions, the coordinates corresponding to BCAFV peaks are more consistent with the spatial location relationship of four antennas. The number of the valid epoch is increased from 69.21% to 84.16% after replacing the float solutions with the coordinates corresponding to BCAFV peaks. The precision of the heading angle is 0.54°, while the pitch and roll angles are 1.46° and 1.15°, respectively. The RMS of known baseline residuals is 3.8 mm.

## 5. Discussion

The GNSS attitude determination algorithm has been very mature, and it can be realized by using three GNSS antennas to form two independent baselines. Theoretically, a baseline of 1 m length can obtain an accuracy of 0.6° and the longer the baseline, the higher the accuracy [38]. The baseline length in our platform is 0.3 m and 0.424 m. The precision of attitude determination is consistent with the theory. However, GNSS attitude determination depends on the accuracy of antenna position calculation (baseline vector). Once the ambiguity is not fixed or fixed incorrectly, the result cannot be used completely, which is also the biggest factor restricting the GNSS attitude determination application. Although many researchers have proposed various methods such as reliability tests and multi-baseline assistance, they have not given full play to the role of each available baseline. Multi-antennas can not only supplement the positioning and attitude determination results but also have a certain value in reliability guarantee. This paper is aimed at the above problems. However, there are still some shortcomings in this paper. For example, the difference between the known and calculated length by the proposed method will be very large if the ambiguity is incorrectly fixed. To avoid a large number of wrong attitude information, we do not calculate them under these circumstances. Receiver Autonomous Integrity Monitoring (RAIM) or other quality control ideas can be introduced. With the help of these methods, the specific reasons why ambiguity is not fixed can be found and solved in time. In addition, the efficiency of AFM can be further improved by Particle swarm optimization (PSO) and other optimization algorithms. If the reliable attitude of every epoch still cannot be obtained, such as Case 5, the low-cost MEMS IMU chip can be considered to integrate to supplement attitude results when GNSS is not available. In addition, due to its small size, this platform will be built on unmanned aerial vehicles in the future. All these problems are worth further discussion and study.

## Figures and Tables

**Table 1:** The number of epochs corresponding to five cases.

Case	1	2	3	4	5	Total Epochs
Number	616	55	76	2	141	890

**Table 2:** The search time of AFM and BCAFM in three sample epochs.

Epoch	GPST	Traditional AFM	BCAFM
1	03:03:31	1.9720	0.2836
2	03:05:15	2.6213	0.3410
3	03:03:37	2.5650	0.3290

**Table 3:** The Ambiguity resolutions of four antennas in the selected three epochs.

GPST	The Categories of Cases in Table 1	Ambiguity Resolutions of Four Antennas			
		1	2	3	4
03:03:31	Case 2	Fixed	Fixed	Float	Fixed
03:05:15	Case 3	Fixed	Float	Float	Fixed
03:03:37	Case 4	Fixed	Float	Float	Float

**Table 4:** Several large AFVs and BCAFVs and corresponding coordinates in epoch 03:03:31.

X (m)	Y (m)	Z (m)	AFV	Remark	X (m)	Y (m)	Z (m)	BCAFV	Remark
06.80	38.98	16.70	0.8895	Peak1	06.88	39.12	15.98	0.8249	Peak2
06.78	38.98	16.70	0.8704		06.86	39.12	16.00	0.8036	
06.78	38.94	16.70	0.8482		06.88	39.14	16.00	0.7932	
06.88	39.12	15.98	0.8331	Peak2	06.88	39.12	15.98	0.7748	
06.86	39.12	16.00	0.8196		06.86	39.12	15.98	0.7720	
06.78	38.92	16.70	0.7917		06.86	39.14	16.00	0.7702	

**Table 5:** Several large AFVs and BCAFVs and corresponding coordinates in epoch 03:05:15.

X (m)	Y (m)	Z (m)	AFV	BCAFV	Remark
46.72	79.10	38.66	0.9723	0.9716	Peak1
46.72	79.10	38.68	0.9695	0.9519	
46.74	79.10	38.66	0.9318	0.9296	
46.72	78.60	38.68	0.9198	0.7913	Peak2
46.72	79.08	38.68	0.8889	0.8651	
46.72	78.60	38.70	0.8877	0.7544	

**Table 6:** Several large AFVs and BCAFVs and corresponding coordinates in epoch 03:03:37.

X (m)	Y (m)	Z (m)	AFV	BCAFV	Remark
74.92	84.71	49.92	0.9305	0.9277	Peak 1
74.94	84.71	49.92	0.9053	0.9027	
74.92	84.73	49.92	0.8977	0.8931	
74.92	84.69	49.92	0.8690	0.8512	
74.94	84.69	49.92	0.8451	0.8444	
74.92	84.75	49.94	0.8350	0.8263	

**Table 7:** The Precision of attitude determination based on BCAFM and RTK.

Method	Precision of Attitude Determination (°)		
	Heading	Pitch	Roll
RTK (fixed and float)	34.23	11.78	19.69
BCAFM	0.54	1.46	1.15

## Data Availability

The data that support the findings of this study are available from the first and corresponding author, upon reasonable request.
